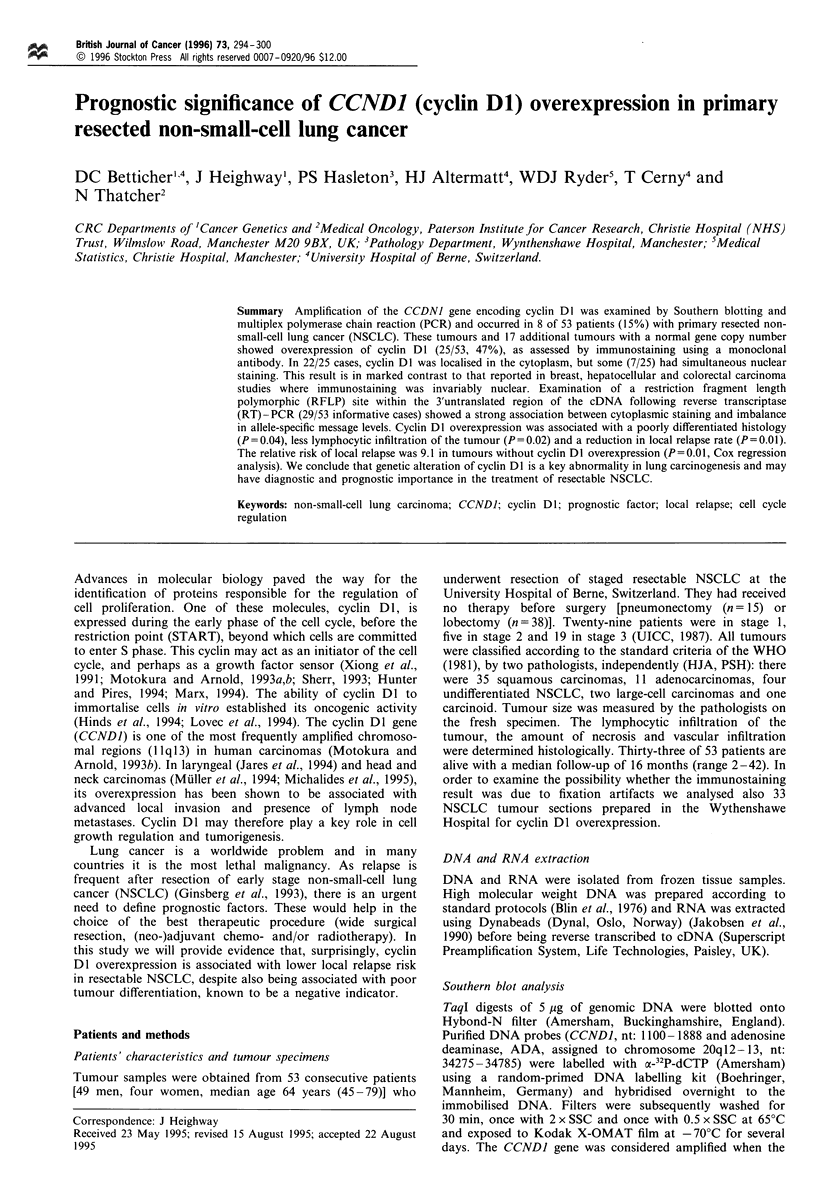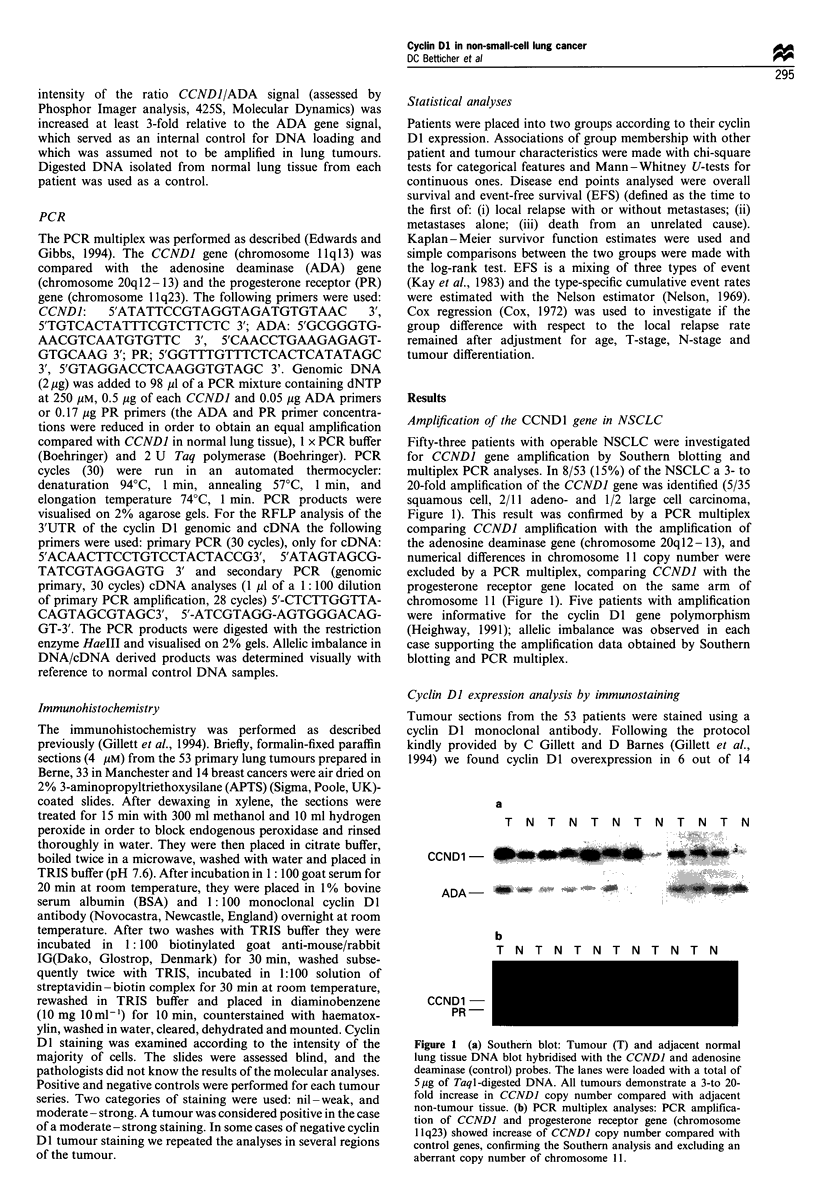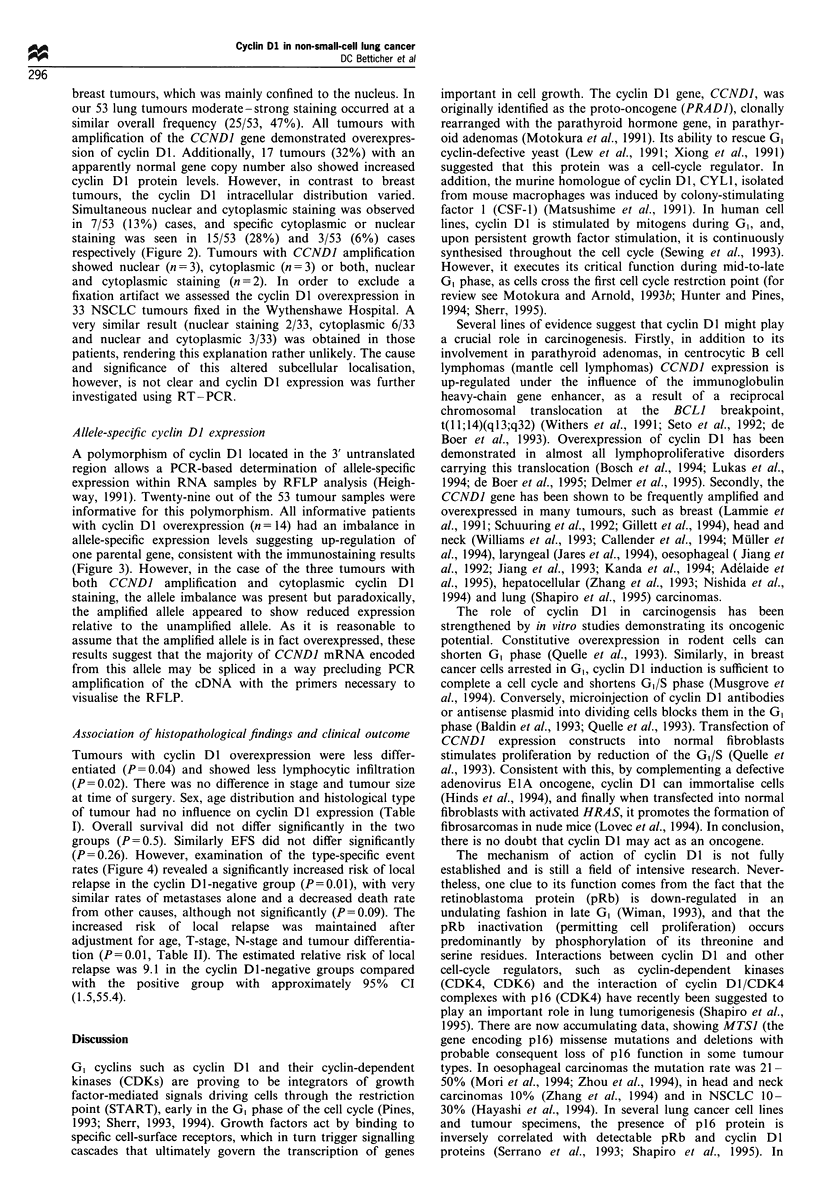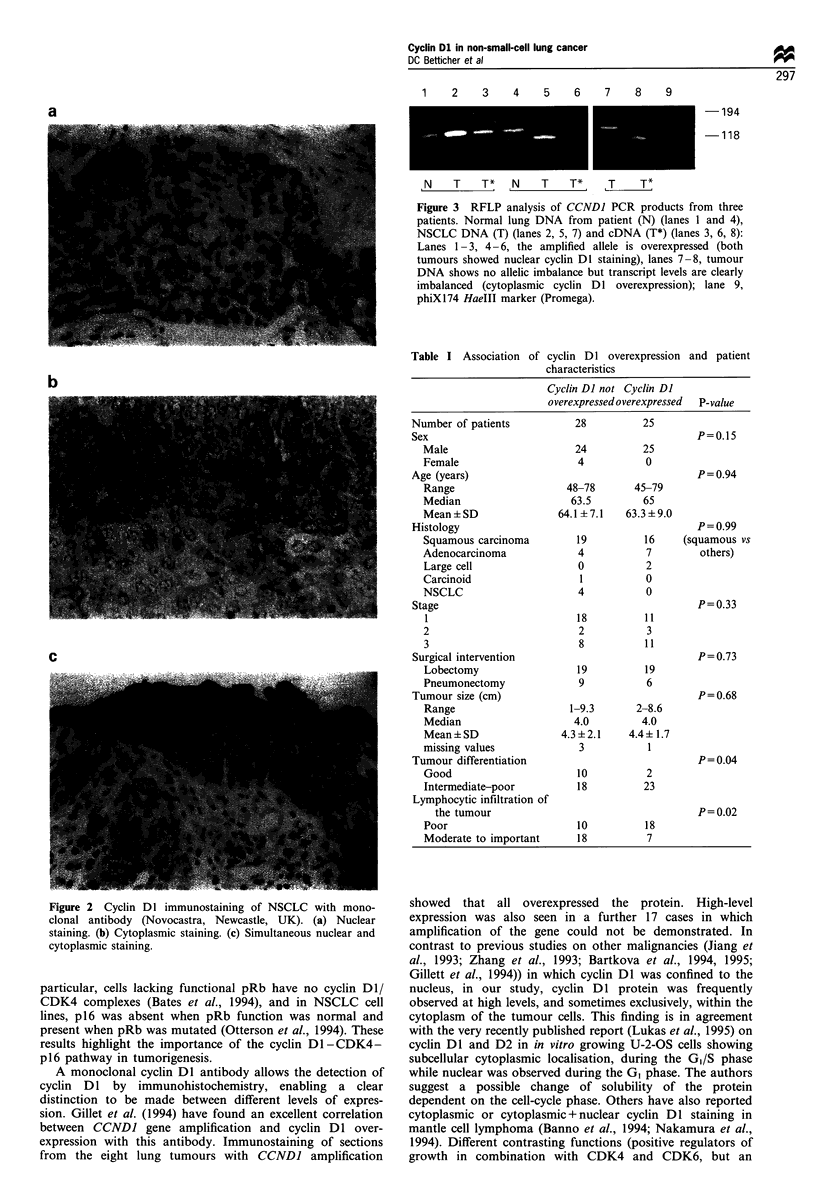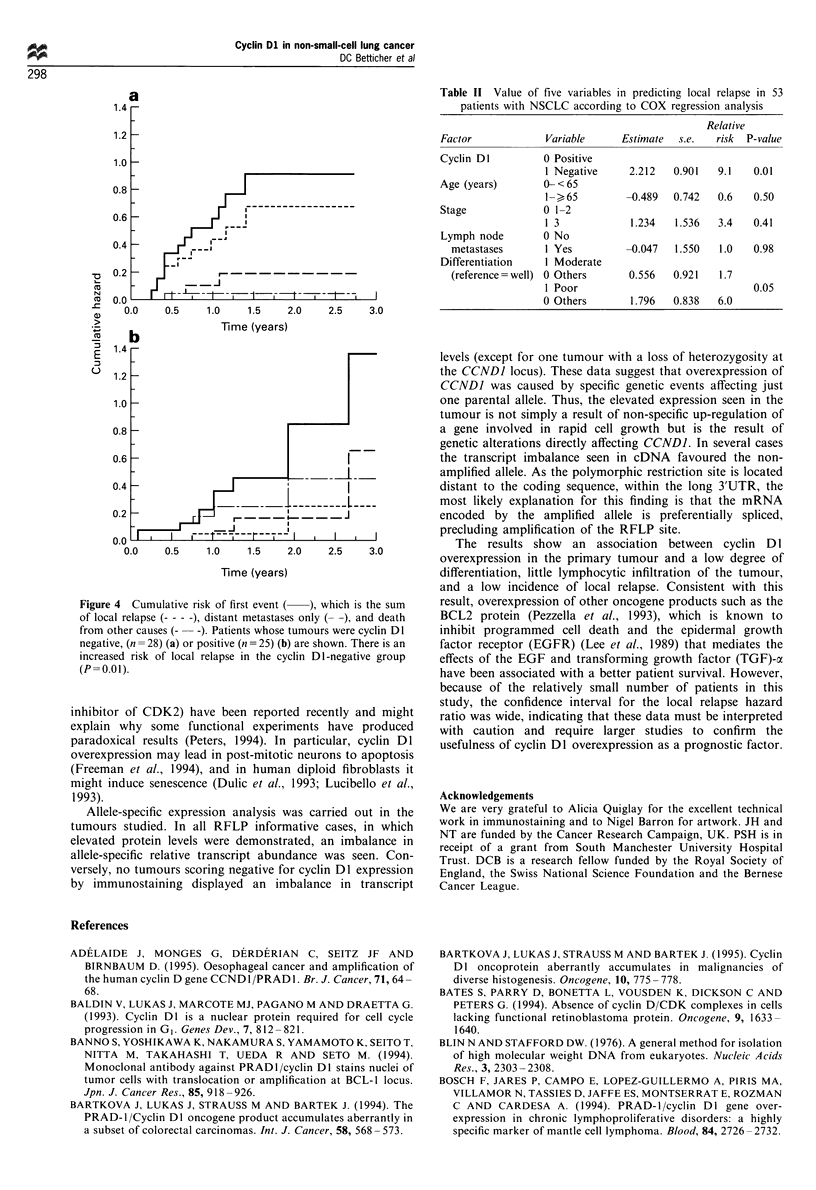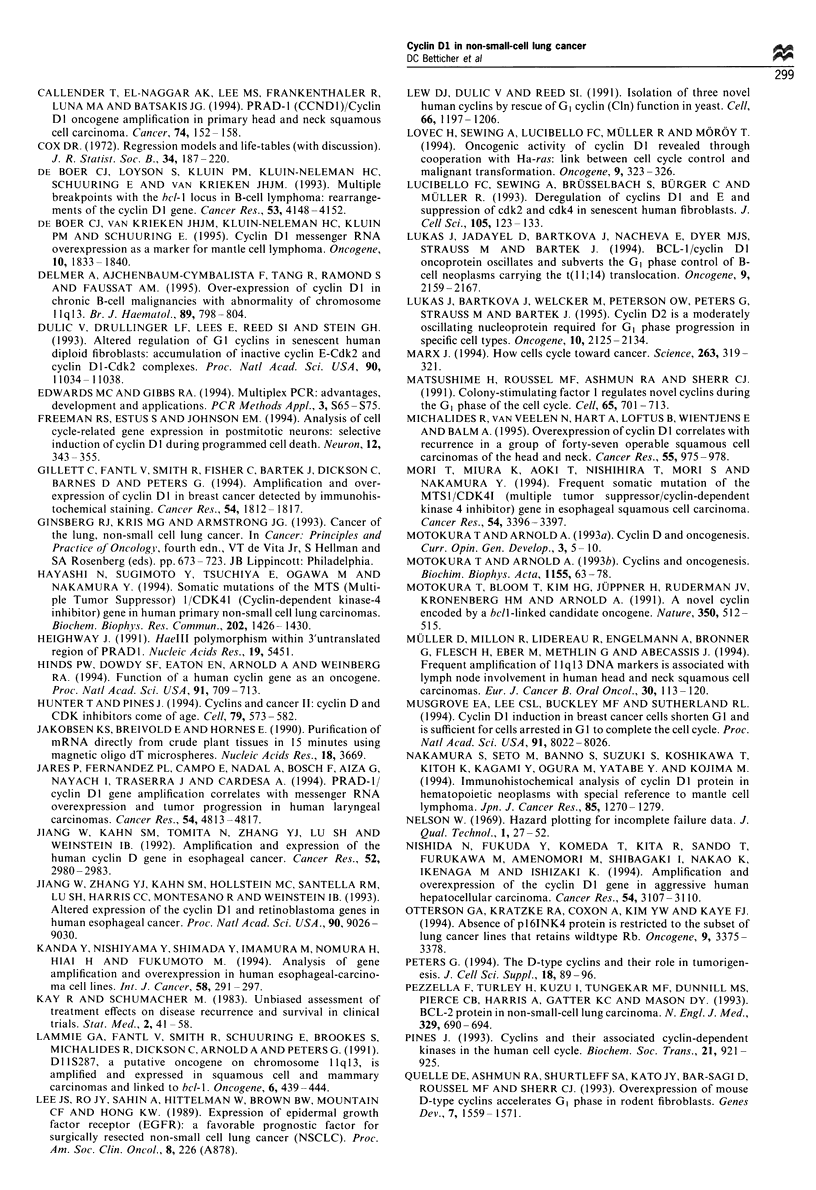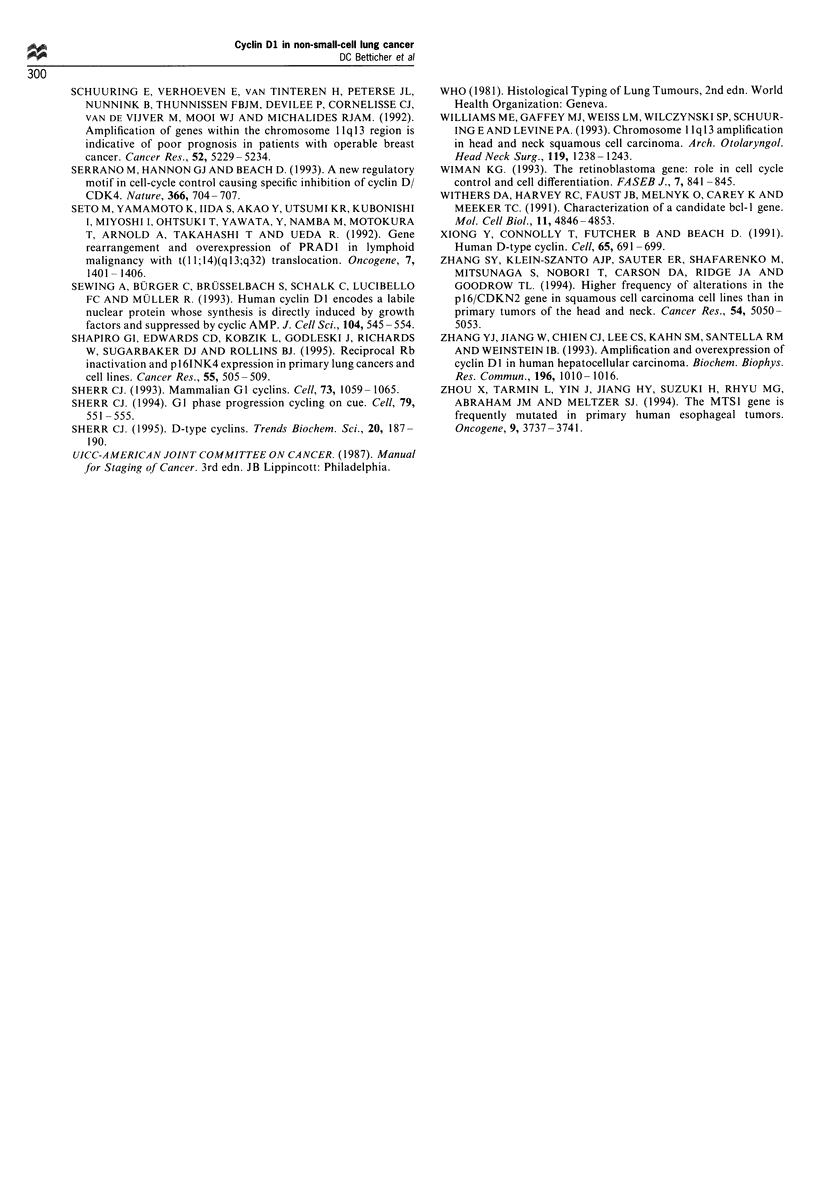# Prognostic significance of CCND1 (cyclin D1) overexpression in primary resected non-small-cell lung cancer.

**DOI:** 10.1038/bjc.1996.52

**Published:** 1996-02

**Authors:** D. C. Betticher, J. Heighway, P. S. Hasleton, H. J. Altermatt, W. D. Ryder, T. Cerny, N. Thatcher

**Affiliations:** CRC Department of Cancer Genetics, Paterson Institute for Cancer Research, Christie Hospital (NHS) Trust, Manchester, UK.

## Abstract

**Images:**


British Journal of Cancer (1996) 73, 294-300
© 1996 Stockton Press All rights reserved 0007-0920/96 $12.00

### Prognostic significance of *CCND1* (cyclin D1) overexpression in primary resected non-small-cell lung cancer

DC Betticher[1,4], J Heighway[1], PS Hasleton[3], HJ Altermatt[4], WDJ Ryder[5], T Cerny[4] and N Thatcher[2]

*CRC Departments of [1]Cancer Genetics and [2]Medical Oncology, Paterson Institute for Cancer Research, Christie Hospital (NHS) Trust, Wilmslow Road, Manchester M20 9BX, UK; [3]Pathology Department, Wynthenshawe Hospital, Manchester; [5]Medical Statistics, Christie Hospital, Manchester; [4]University Hospital of Berne, Switzerland.*

**Summary** Amplification of the *CCDN1* gene encoding cyclin D1 was examined by Southern blotting and multiplex polymerase chain reaction (PCR) and occurred in 8 of 53 patients (15%) with primary resected non-small-cell lung cancer (NSCLC). These tumours and 17 additional tumours with a normal gene copy number showed overexpression of cyclin D1 (25/53, 47%), as assessed by immunostaining using a monoclonal antibody. In 22/25 cases, cyclin D1 was localised in the cytoplasm, but some (7/25) had simultaneous nuclear staining. This result is in marked contrast to that reported in breast, hepatocellular and colorectal carcinoma studies where immunostaining was invariably nuclear. Examination of a restriction fragment length polymorphic (RFLP) site within the 3'untranslated region of the cDNA following reverse transcriptase (RT)-PCR (29/53 informative cases) showed a strong association between cytoplasmic staining and imbalance in allele-specific message levels. Cyclin D1 overexpression was associated with a poorly differentiated histology ($P=0.04$), less lymphocytic infiltration of the tumour ($P=0.02$) and a reduction in local relapse rate ($P=0.01$). The relative risk of local relapse was 9.1 in tumours without cyclin D1 overexpression ($P=0.01$, Cox regression analysis). We conclude that genetic alteration of cyclin D1 is a key abnormality in lung carcinogenesis and may have diagnostic and prognostic importance in the treatment of resectable NSCLC.

**Keywords:** non-small-cell lung carcinoma; *CCND1*; cyclin D1; prognostic factor; local relapse; cell cycle regulation

Advances in molecular biology paved the way for the identification of proteins responsible for the regulation of cell proliferation. One of these molecules, cyclin D1, is expressed during the early phase of the cell cycle, before the restriction point (START), beyond which cells are committed to enter S phase. This cyclin may act as an initiator of the cell cycle, and perhaps as a growth factor sensor (Xiong *et al.*, 1991; Motokura and Arnold, 1993*a*,*b*; Sherr, 1993; Hunter and Pires, 1994; Marx, 1994). The ability of cyclin D1 to immortalise cells *in vitro* established its oncogenic activity (Hinds *et al.*, 1994; Lovec *et al.*, 1994). The cyclin D1 gene (*CCND1*) is one of the most frequently amplified chromosomal regions (11q13) in human carcinomas (Motokura and Arnold, 1993*b*). In laryngeal (Jares *et al.*, 1994) and head and neck carcinomas (Müller *et al.*, 1994; Michalides *et al.*, 1995), its overexpression has been shown to be associated with advanced local invasion and presence of lymph node metastases. Cyclin D1 may therefore play a key role in cell growth regulation and tumorigenesis.

Lung cancer is a worldwide problem and in many countries it is the most lethal malignancy. As relapse is frequent after resection of early stage non-small-cell lung cancer (NSCLC) (Ginsberg *et al.*, 1993), there is an urgent need to define prognostic factors. These would help in the choice of the best therapeutic procedure (wide surgical resection, (neo-)adjuvant chemo- and/or radiotherapy). In this study we will provide evidence that, surprisingly, cyclin D1 overexpression is associated with lower local relapse risk in resectable NSCLC, despite also being associated with poor tumour differentiation, known to be a negative indicator.

### Patients and methods

### *Patients' characteristics and tumour specimens*

Tumour samples were obtained from 53 consecutive patients [49 men, four women, median age 64 years (45-79)] who underwent resection of staged resectable NSCLC at the University Hospital of Berne, Switzerland. They had received no therapy before surgery [pneumonectomy ($n=15$) or lobectomy ($n=38$)]. Twenty-nine patients were in stage 1, five in stage 2 and 19 in stage 3 (UICC, 1987). All tumours were classified according to the standard criteria of the WHO (1981), by two pathologists, independently (HJA, PSH): there were 35 squamous carcinomas, 11 adenocarcinomas, four undifferentiated NSCLC, two large-cell carcinomas and one carcinoid. Tumour size was measured by the pathologists on the fresh specimen. The lymphocytic infiltration of the tumour, the amount of necrosis and vascular infiltration were determined histologically. Thirty-three of 53 patients are alive with a median follow-up of 16 months (range 2-42). In order to examine the possibility whether the immunostaining result was due to fixation artifacts we analysed also 33 NSCLC tumour sections prepared in the Wythenshawe Hospital for cyclin D1 overexpression.

### *DNA and RNA extraction*

DNA and RNA were isolated from frozen tissue samples. High molecular weight DNA was prepared according to standard protocols (Blin *et al.*, 1976) and RNA was extracted using Dynabeads (Dynal, Oslo, Norway) (Jakobsen *et al.*, 1990) before being reverse transcribed to cDNA (Superscript Preamplification System, Life Technologies, Paisley, UK).

### *Southern blot analysis*

*Taq*I digests of 5 μg of genomic DNA were blotted onto Hybond-N filter (Amersham, Buckinghamshire, England). Purified DNA probes (*CCND1*, nt: 1100-1888 and adenosine deaminase, ADA, assigned to chromosome 20q12-13, nt: 34275-34785) were labelled with $\alpha$-$^{32}$P-dCTP (Amersham) using a random-primed DNA labelling kit (Boehringer, Mannheim, Germany) and hybridised overnight to the immobilised DNA. Filters were subsequently washed for 30 min, once with $2\times$SSC and once with $0.5\times$SSC at 65°C and exposed to Kodak X-OMAT film at $-70$°C for several days. The *CCND1* gene was considered amplified when the

Correspondence: J Heighway
Received 23 May 1995; revised 15 August 1995; accepted 22 August 1995

intensity of the ratio *CCND1*/ADA signal (assessed by Phosphor Imager analysis, 425S, Molecular Dynamics) was increased at least 3-fold relative to the ADA gene signal, which served as an internal control for DNA loading and which was assumed not to be amplified in lung tumours. Digested DNA isolated from normal lung tissue from each patient was used as a control.

*PCR*

The PCR multiplex was performed as described (Edwards and Gibbs, 1994). The *CCND1* gene (chromosome 11q13) was compared with the adenosine deaminase (ADA) gene (chromosome 20q12–13) and the progesterone receptor (PR) gene (chromosome 11q23). The following primers were used: *CCND1*: 5'ATATTCCGTAGGTAGATGTGTAAC 3', 5'TGTCACTATTTCGTCTTCTC 3'; ADA: 5'GCGGGTG-AACGTCAATGTGTTC 3', 5'CAACCTGAAGAGAGT-GTGCAAG 3'; PR; 5'GGTTTGTTTCTCACTCATATAGC 3', 5'GTAGGACCTCAAGGTGTAGC 3'. Genomic DNA (2 $\mu$g) was added to 98 $\mu$l of a PCR mixture containing dNTP at 250 $\mu$M, 0.5 $\mu$g of each *CCND1* and 0.05 $\mu$g ADA primers or 0.17 $\mu$g PR primers (the ADA and PR primer concentrations were reduced in order to obtain an equal amplification compared with *CCND1* in normal lung tissue), 1 × PCR buffer (Boehringer) and 2 U *Taq* polymerase (Boehringer). PCR cycles (30) were run in an automated thermocycler: denaturation 94°C, 1 min, annealing 57°C, 1 min, and elongation temperature 74°C, 1 min. PCR products were visualised on 2% agarose gels. For the RFLP analysis of the 3'UTR of the cyclin D1 genomic and cDNA the following primers were used: primary PCR (30 cycles), only for cDNA: 5'ACAACTTCCTGTCCTACTACCG3', 5'ATAGTAGCG-TATCGTAGGAGTG 3' and secondary PCR (genomic primary, 30 cycles) cDNA analyses (1 $\mu$l of a 1 : 100 dilution of primary PCR amplification, 28 cycles) 5'-CTCTTGGTTA-CAGTAGCGTAGC3', 5'-ATCGTAGG-AGTGGGACAG-GT-3'. The PCR products were digested with the restriction enzyme *Hae*III and visualised on 2% gels. Allelic imbalance in DNA/cDNA derived products was determined visually with reference to normal control DNA samples.

*Immunohistochemistry*

The immunohistochemistry was performed as described previously (Gillett *et al*., 1994). Briefly, formalin-fixed paraffin sections (4 $\mu$M) from the 53 primary lung tumours prepared in Berne, 33 in Manchester and 14 breast cancers were air dried on 2% 3-aminopropyltriethoxysilane (APTS) (Sigma, Poole, UK)-coated slides. After dewaxing in xylene, the sections were treated for 15 min with 300 ml methanol and 10 ml hydrogen peroxide in order to block endogenous peroxidase and rinsed thoroughly in water. They were then placed in citrate buffer, boiled twice in a microwave, washed with water and placed in TRIS buffer (pH 7.6). After incubation in 1 : 100 goat serum for 20 min at room temperature, they were placed in 1% bovine serum albumin (BSA) and 1 : 100 monoclonal cyclin D1 antibody (Novocastra, Newcastle, England) overnight at room temperature. After two washes with TRIS buffer they were incubated in 1 : 100 biotinylated goat anti-mouse/rabbit IG(Dako, Glostrop, Denmark) for 30 min, washed subsequently twice with TRIS, incubated in 1:100 solution of streptavidin–biotin complex for 30 min at room temperature, rewashed in TRIS buffer and placed in diaminobenzene (10 mg 10 ml$^{-1}$) for 10 min, counterstained with haematoxylin, washed in water, cleared, dehydrated and mounted. Cyclin D1 staining was examined according to the intensity of the majority of cells. The slides were assessed blind, and the pathologists did not know the results of the molecular analyses. Positive and negative controls were performed for each tumour series. Two categories of staining were used: nil–weak, and moderate–strong. A tumour was considered positive in the case of a moderate–strong staining. In some cases of negative cyclin D1 tumour staining we repeated the analyses in several regions of the tumour.

*Statistical analyses*

Patients were placed into two groups according to their cyclin D1 expression. Associations of group membership with other patient and tumour characteristics were made with chi-square tests for categorical features and Mann–Whitney *U*-tests for continuous ones. Disease end points analysed were overall survival and event-free survival (EFS) (defined as the time to the first of: (i) local relapse with or without metastases; (ii) metastases alone; (iii) death from an unrelated cause). Kaplan–Meier survivor function estimates were used and simple comparisons between the two groups were made with the log-rank test. EFS is a mixing of three types of event (Kay *et al*., 1983) and the type-specific cumulative event rates were estimated with the Nelson estimator (Nelson, 1969). Cox regression (Cox, 1972) was used to investigate if the group difference with respect to the local relapse rate remained after adjustment for age, T-stage, N-stage and tumour differentiation.

### Results

*Amplification of the* CCND1 *gene in NSCLC*

Fifty-three patients with operable NSCLC were investigated for *CCND1* gene amplification by Southern blotting and multiplex PCR analyses. In 8/53 (15%) of the NSCLC a 3- to 20-fold amplification of the *CCND1* gene was identified (5/35 squamous cell, 2/11 adeno- and 1/2 large cell carcinoma, Figure 1). This result was confirmed by a PCR multiplex comparing *CCND1* amplification with the amplification of the adenosine deaminase gene (chromosome 20q12–13), and numerical differences in chromosome 11 copy number were excluded by a PCR multiplex, comparing *CCND1* with the progesterone receptor gene located on the same arm of chromosome 11 (Figure 1). Five patients with amplification were informative for the cyclin D1 gene polymorphism (Heighway, 1991); allelic imbalance was observed in each case supporting the amplification data obtained by Southern blotting and PCR multiplex.

*Cyclin D1 expression analysis by immunostaining*

Tumour sections from the 53 patients were stained using a cyclin D1 monoclonal antibody. Following the protocol kindly provided by C Gillett and D Barnes (Gillett *et al*., 1994) we found cyclin D1 overexpression in 6 out of 14

**Figure 1** (**a**) Southern blot: Tumour (T) and adjacent normal lung tissue DNA blot hybridised with the *CCND1* and adenosine deaminase (control) probes. The lanes were loaded with a total of 5 $\mu$g of *Taq*1-digested DNA. All tumours demonstrate a 3-to 20-fold increase in *CCND1* copy number compared with adjacent non-tumour tissue. (**b**) PCR multiplex analyses: PCR amplification of *CCND1* and progesterone receptor gene (chromosome 11q23) showed increase of *CCND1* copy number compared with control genes, confirming the Southern analysis and excluding an aberrant copy number of chromosome 11.

breast tumours, which was mainly confined to the nucleus. In our 53 lung tumours moderate–strong staining occurred at a similar overall frequency (25/53, 47%). All tumours with amplification of the *CCND1* gene demonstrated overexpression of cyclin D1. Additionally, 17 tumours (32%) with an apparently normal gene copy number also showed increased cyclin D1 protein levels. However, in contrast to breast tumours, the cyclin D1 intracellular distribution varied. Simultaneous nuclear and cytoplasmic staining was observed in 7/53 (13%) cases, and specific cytoplasmic or nuclear staining was seen in 15/53 (28%) and 3/53 (6%) cases respectively (Figure 2). Tumours with *CCND1* amplification showed nuclear ($n=3$), cytoplasmic ($n=3$) or both, nuclear and cytoplasmic staining ($n=2$). In order to exclude a fixation artifact we assessed the cyclin D1 overexpression in 33 NSCLC tumours fixed in the Wythenshawe Hospital. A very similar result (nuclear staining 2/33, cytoplasmic 6/33 and nuclear and cytoplasmic 3/33) was obtained in those patients, rendering this explanation rather unlikely. The cause and significance of this altered subcellular localisation, however, is not clear and cyclin D1 expression was further investigated using RT–PCR.

*Allele-specific cyclin D1 expression*

A polymorphism of cyclin D1 located in the 3′ untranslated region allows a PCR-based determination of allele-specific expression within RNA samples by RFLP analysis (Heighway, 1991). Twenty-nine out of the 53 tumour samples were informative for this polymorphism. All informative patients with cyclin D1 overexpression ($n=14$) had an imbalance in allele-specific expression levels suggesting up-regulation of one parental gene, consistent with the immunostaining results (Figure 3). However, in the case of the three tumours with both *CCND1* amplification and cytoplasmic cyclin D1 staining, the allele imbalance was present but paradoxically, the amplified allele appeared to show reduced expression relative to the unamplified allele. As it is reasonable to assume that the amplified allele is in fact overexpressed, these results suggest that the majority of *CCND1* mRNA encoded from this allele may be spliced in a way precluding PCR amplification of the cDNA with the primers necessary to visualise the RFLP.

*Association of histopathological findings and clinical outcome*

Tumours with cyclin D1 overexpression were less differentiated ($P=0.04$) and showed less lymphocytic infiltration ($P=0.02$). There was no difference in stage and tumour size at time of surgery. Sex, age distribution and histological type of tumour had no influence on cyclin D1 expression (Table I). Overall survival did not differ significantly in the two groups ($P=0.5$). Similarly EFS did not differ significantly ($P=0.26$). However, examination of the type-specific event rates (Figure 4) revealed a significantly increased risk of local relapse in the cyclin D1-negative group ($P=0.01$), with very similar rates of metastases alone and a decreased death rate from other causes, although not significantly ($P=0.09$). The increased risk of local relapse was maintained after adjustment for age, T-stage, N-stage and tumour differentiation ($P=0.01$, Table II). The estimated relative risk of local relapse was 9.1 in the cyclin D1-negative groups compared with the positive group with approximately 95% CI (1.5,55.4).

### Discussion

$G_1$ cyclins such as cyclin D1 and their cyclin-dependent kinases (CDKs) are proving to be integrators of growth factor-mediated signals driving cells through the restriction point (START), early in the $G_1$ phase of the cell cycle (Pines, 1993; Sherr, 1993, 1994). Growth factors act by binding to specific cell-surface receptors, which in turn trigger signalling cascades that ultimately govern the transcription of genes important in cell growth. The cyclin D1 gene, *CCND1*, was originally identified as the proto-oncogene (*PRAD1*), clonally rearranged with the parathyroid hormone gene, in parathyroid adenomas (Motokura *et al*., 1991). Its ability to rescue $G_1$ cyclin-defective yeast (Lew *et al*., 1991; Xiong *et al*., 1991) suggested that this protein was a cell-cycle regulator. In addition, the murine homologue of cyclin D1, CYL1, isolated from mouse macrophages was induced by colony-stimulating factor 1 (CSF-1) (Matsushime *et al*., 1991). In human cell lines, cyclin D1 is stimulated by mitogens during $G_1$, and, upon persistent growth factor stimulation, it is continuously synthesised throughout the cell cycle (Sewing *et al*., 1993). However, it executes its critical function during mid-to-late $G_1$ phase, as cells cross the first cell cycle restrction point (for review see Motokura and Arnold, 1993*b*; Hunter and Pines, 1994; Sherr, 1995).

Several lines of evidence suggest that cyclin D1 might play a crucial role in carcinogenesis. Firstly, in addition to its involvement in parathyroid adenomas, in centrocytic B cell lymphomas (mantle cell lymphomas) *CCND1* expression is up-regulated under the influence of the immunoglobulin heavy-chain gene enhancer, as a result of a reciprocal chromosomal translocation at the *BCL1* breakpoint, t(11;14)(q13;q32) (Withers *et al*., 1991; Seto *et al*., 1992; de Boer *et al*., 1993). Overexpression of cyclin D1 has been demonstrated in almost all lymphoproliferative disorders carrying this translocation (Bosch *et al*., 1994; Lukas *et al*., 1994; de Boer *et al*., 1995; Delmer *et al*., 1995). Secondly, the *CCND1* gene has been shown to be frequently amplified and overexpressed in many tumours, such as breast (Lammie *et al*., 1991; Schuuring *et al*., 1992; Gillett *et al*., 1994), head and neck (Williams *et al*., 1993; Callender *et al*., 1994; Müller *et al*., 1994), laryngeal (Jares *et al*., 1994), oesophageal ( Jiang *et al*., 1992; Jiang *et al*., 1993; Kanda *et al*., 1994; Adélaide *et al*., 1995), hepatocellular (Zhang *et al*., 1993; Nishida *et al*., 1994) and lung (Shapiro *et al*., 1995) carcinomas.

The role of cyclin D1 in carcinogensis has been strengthened by *in vitro* studies demonstrating its oncogenic potential. Constitutive overexpression in rodent cells can shorten $G_1$ phase (Quelle *et al*., 1993). Similarly, in breast cancer cells arrested in $G_1$, cyclin D1 induction is sufficient to complete a cell cycle and shortens $G_1/S$ phase (Musgrove *et al*., 1994). Conversely, microinjection of cyclin D1 antibodies or antisense plasmid into dividing cells blocks them in the $G_1$ phase (Baldin *et al*., 1993; Quelle *et al*., 1993). Transfection of *CCND1* expression constructs into normal fibroblasts stimulates proliferation by reduction of the $G_1/S$ (Quelle *et al*., 1993). Consistent with this, by complementing a defective adenovirus E1A oncogene, cyclin D1 can immortalise cells (Hinds *et al*., 1994), and finally when transfected into normal fibroblasts with activated *HRAS*, it promotes the formation of fibrosarcomas in nude mice (Lovec *et al*., 1994). In conclusion, there is no doubt that cyclin D1 may act as an oncogene.

The mechanism of action of cyclin D1 is not fully established and is still a field of intensive research. Nevertheless, one clue to its function comes from the fact that the retinoblastoma protein (pRb) is down-regulated in an undulating fashion in late $G_1$ (Wiman, 1993), and that the pRb inactivation (permitting cell proliferation) occurs predominantly by phosphorylation of its threonine and serine residues. Interactions between cyclin D1 and other cell-cycle regulators, such as cyclin-dependent kinases (CDK4, CDK6) and the interaction of cyclin D1/CDK4 complexes with p16 (CDK4) have recently been suggested to play an important role in lung tumorigenesis (Shapiro *et al*., 1995). There are now accumulating data, showing *MTS1* (the gene encoding p16) missense mutations and deletions with probable consequent loss of p16 function in some tumour types. In oesophageal carcinomas the mutation rate was 21–50% (Mori *et al*., 1994; Zhou *et al*., 1994), in head and neck carcinomas 10% (Zhang *et al*., 1994) and in NSCLC 10–30% (Hayashi *et al*., 1994). In several lung cancer cell lines and tumour specimens, the presence of p16 protein is inversely correlated with detectable pRb and cyclin D1 proteins (Serrano *et al*., 1993; Shapiro *et al*., 1995). In

**Figure 2** Cyclin D1 immunostaining of NSCLC with monoclonal antibody (Novocastra, Newcastle, UK). (**a**) Nuclear staining. (**b**) Cytoplasmic staining. (**c**) Simultaneous nuclear and cytoplasmic staining.

**Figure 3** RFLP analysis of *CCND1* PCR products from three patients. Normal lung DNA from patient (N) (lanes 1 and 4), NSCLC DNA (T) (lanes 2, 5, 7) and cDNA (T*) (lanes 3, 6, 8): Lanes 1–3, 4–6, the amplified allele is overexpressed (both tumours showed nuclear cyclin D1 staining), lanes 7–8, tumour DNA shows no allelic imbalance but transcript levels are clearly imbalanced (cytoplasmic cyclin D1 overexpression); lane 9, phiX174 *Hae*III marker (Promega).

**Table I** Association of cyclin D1 overexpression and patient characteristics

| | *Cyclin D1 not overexpressed* | *Cyclin D1 overexpressed* | P-*value* |
|---|---|---|---|
| Number of patients | 28 | 25 | |
| Sex | | | $P = 0.15$ |
| Male | 24 | 25 | |
| Female | 4 | 0 | |
| Age (years) | | | $P = 0.94$ |
| Range | 48–78 | 45–79 | |
| Median | 63.5 | 65 | |
| Mean ± SD | 64.1 ± 7.1 | 63.3 ± 9.0 | |
| Histology | | | $P = 0.99$ |
| Squamous carcinoma | 19 | 16 | (squamous *vs* |
| Adenocarcinoma | 4 | 7 | others) |
| Large cell | 0 | 2 | |
| Carcinoid | 1 | 0 | |
| NSCLC | 4 | 0 | |
| Stage | | | $P = 0.33$ |
| 1 | 18 | 11 | |
| 2 | 2 | 3 | |
| 3 | 8 | 11 | |
| Surgical intervention | | | $P = 0.73$ |
| Lobectomy | 19 | 19 | |
| Pneumonectomy | 9 | 6 | |
| Tumour size (cm) | | | $P = 0.68$ |
| Range | 1–9.3 | 2–8.6 | |
| Median | 4.0 | 4.0 | |
| Mean ± SD | 4.3 ± 2.1 | 4.4 ± 1.7 | |
| missing values | 3 | 1 | |
| Tumour differentiation | | | $P = 0.04$ |
| Good | 10 | 2 | |
| Intermediate–poor | 18 | 23 | |
| Lymphocytic infiltration of the tumour | | | $P = 0.02$ |
| Poor | 10 | 18 | |
| Moderate to important | 18 | 7 | |

particular, cells lacking functional pRb have no cyclin D1/CDK4 complexes (Bates *et al.*, 1994), and in NSCLC cell lines, p16 was absent when pRb function was normal and present when pRb was mutated (Otterson *et al.*, 1994). These results highlight the importance of the cyclin D1–CDK4–p16 pathway in tumorigenesis.

A monoclonal cyclin D1 antibody allows the detection of cyclin D1 by immunohistochemistry, enabling a clear distinction to be made between different levels of expression. Gillet *et al.* (1994) have found an excellent correlation between *CCND1* gene amplification and cyclin D1 overexpression with this antibody. Immunostaining of sections from the eight lung tumours with *CCND1* amplification showed that all overexpressed the protein. High-level expression was also seen in a further 17 cases in which amplification of the gene could not be demonstrated. In contrast to previous studies on other malignancies (Jiang *et al.*, 1993; Zhang *et al.*, 1993; Bartkova *et al.*, 1994, 1995; Gillett *et al.*, 1994)) in which cyclin D1 was confined to the nucleus, in our study, cyclin D1 protein was frequently observed at high levels, and sometimes exclusively, within the cytoplasm of the tumour cells. This finding is in agreement with the very recently published report (Lukas *et al.*, 1995) on cyclin D1 and D2 in *in vitro* growing U-2-OS cells showing subcellular cytoplasmic localisation, during the $G_1/S$ phase while nuclear was observed during the $G_1$ phase. The authors suggest a possible change of solubility of the protein dependent on the cell-cycle phase. Others have also reported cytoplasmic or cytoplasmic + nuclear cyclin D1 staining in mantle cell lymphoma (Banno *et al.*, 1994; Nakamura *et al.*, 1994). Different contrasting functions (positive regulators of growth in combination with CDK4 and CDK6, but an

**Figure 4** Cumulative risk of first event (——), which is the sum of local relapse (- - - -), distant metastases only (– –), and death from other causes (- — -). Patients whose tumours were cyclin D1 negative, ($n=28$) (**a**) or positive ($n=25$) (**b**) are shown. There is an increased risk of local relapse in the cyclin D1-negative group ($P=0.01$).

**Table II** Value of five variables in predicting local relapse in 53 patients with NSCLC according to COX regression analysis

| *Factor* | *Variable* | *Estimate* | *s.e.* | *Relative risk* | P-*value* |
|---|---|---|---|---|---|
| Cyclin D1 | 0 Positive | | | | |
| | 1 Negative | 2.212 | 0.901 | 9.1 | 0.01 |
| Age (years) | 0– < 65 | | | | |
| | 1– ≥ 65 | –0.489 | 0.742 | 0.6 | 0.50 |
| Stage | 0 1–2 | | | | |
| | 1 3 | 1.234 | 1.536 | 3.4 | 0.41 |
| Lymph node | 0 No | | | | |
| metastases | 1 Yes | –0.047 | 1.550 | 1.0 | 0.98 |
| Differentiation | 1 Moderate | | | | |
| (reference = well) | 0 Others | 0.556 | 0.921 | 1.7 | |
| | 1 Poor | | | | 0.05 |
| | 0 Others | 1.796 | 0.838 | 6.0 | |

inhibitor of CDK2) have been reported recently and might explain why some functional experiments have produced paradoxical results (Peters, 1994). In particular, cyclin D1 overexpression may lead in post-mitotic neurons to apoptosis (Freeman *et al.*, 1994), and in human diploid fibroblasts it might induce senescence (Dulic *et al.*, 1993; Lucibello *et al.*, 1993).

Allele-specific expression analysis was carried out in the tumours studied. In all RFLP informative cases, in which elevated protein levels were demonstrated, an imbalance in allele-specific relative transcript abundance was seen. Conversely, no tumours scoring negative for cyclin D1 expression by immunostaining displayed an imbalance in transcript levels (except for one tumour with a loss of heterozygosity at the *CCND1* locus). These data suggest that overexpression of *CCND1* was caused by specific genetic events affecting just one parental allele. Thus, the elevated expression seen in the tumour is not simply a result of non-specific up-regulation of a gene involved in rapid cell growth but is the result of genetic alterations directly affecting *CCND1*. In several cases the transcript imbalance seen in cDNA favoured the non-amplified allele. As the polymorphic restriction site is located distant to the coding sequence, within the long 3′UTR, the most likely explanation for this finding is that the mRNA encoded by the amplified allele is preferentially spliced, precluding amplification of the RFLP site.

The results show an association between cyclin D1 overexpression in the primary tumour and a low degree of differentiation, little lymphocytic infiltration of the tumour, and a low incidence of local relapse. Consistent with this result, overexpression of other oncogene products such as the BCL2 protein (Pezzella *et al.*, 1993), which is known to inhibit programmed cell death and the epidermal growth factor receptor (EGFR) (Lee *et al.*, 1989) that mediates the effects of the EGF and transforming growth factor (TGF)-α have been associated with a better patient survival. However, because of the relatively small number of patients in this study, the confidence interval for the local relapse hazard ratio was wide, indicating that these data must be interpreted with caution and require larger studies to confirm the usefulness of cyclin D1 overexpression as a prognostic factor.

**Acknowledgements**
We are very grateful to Alicia Quiglay for the excellent technical work in immunostaining and to Nigel Barron for artwork. JH and NT are funded by the Cancer Research Campaign, UK. PSH is in receipt of a grant from South Manchester University Hospital Trust. DCB is a research fellow funded by the Royal Society of England, the Swiss National Science Foundation and the Bernese Cancer League.

### References

ADÉLAIDE J, MONGES G, DÉRDÉRIAN C, SEITZ JF AND BIRNBAUM D. (1995). Oesophageal cancer and amplification of the human cyclin D gene CCND1/PRAD1. *Br. J. Cancer*, **71,** 64–68.

BALDIN V, LUKAS J, MARCOTE MJ, PAGANO M AND DRAETTA G. (1993). Cyclin D1 is a nuclear protein required for cell cycle progression in $G_1$. *Genes Dev.*, **7,** 812–821.

BANNO S, YOSHIKAWA K, NAKAMURA S, YAMAMOTO K, SEITO T, NITTA M, TAKAHASHI T, UEDA R AND SETO M. (1994). Monoclonal antibody against PRAD1/cyclin D1 stains nuclei of tumor cells with translocation or amplification at BCL-1 locus. *Jpn. J. Cancer Res.*, **85,** 918–926.

BARTKOVA J, LUKAS J, STRAUSS M AND BARTEK J. (1994). The PRAD-1/Cyclin D1 oncogene product accumulates aberrantly in a subset of colorectal carcinomas. *Int. J. Cancer*, **58,** 568–573.

BARTKOVA J, LUKAS J, STRAUSS M AND BARTEK J. (1995). Cyclin D1 oncoprotein aberrantly accumulates in malignancies of diverse histogenesis. *Oncogene*, **10,** 775–778.

BATES S, PARRY D, BONETTA L, VOUSDEN K, DICKSON C AND PETERS G. (1994). Absence of cyclin D/CDK complexes in cells lacking functional retinoblastoma protein. *Oncogene*, **9,** 1633–1640.

BLIN N AND STAFFORD DW. (1976). A general method for isolation of high molecular weight DNA from eukaryotes. *Nucleic Acids Res.*, **3,** 2303–2308.

BOSCH F, JARES P, CAMPO E, LOPEZ-GUILLERMO A, PIRIS MA, VILLAMOR N, TASSIES D, JAFFE ES, MONTSERRAT E, ROZMAN C AND CARDESA A. (1994). PRAD-1/cyclin D1 gene overexpression in chronic lymphoproliferative disorders: a highly specific marker of mantle cell lymphoma. *Blood*, **84,** 2726–2732.

CALLENDER T, EL-NAGGAR AK, LEE MS, FRANKENTHALER R, LUNA MA AND BATSAKIS JG. (1994). PRAD-1 (CCND1)/Cyclin D1 oncogene amplification in primary head and neck squamous cell carcinoma. *Cancer*, **74**, 152–158.

COX DR. (1972). Regression models and life-tables (with discussion). *J. R. Statist. Soc. B.*, **34**, 187–220.

DE BOER CJ, LOYSON S, KLUIN PM, KLUIN-NELEMAN HC, SCHUURING E AND VAN KRIEKEN JHJM. (1993). Multiple breakpoints with the *bcl*-1 locus in B-cell lymphoma: rearrangements of the cyclin D1 gene. *Cancer Res.*, **53**, 4148–4152.

DE BOER CJ, VAN KRIEKEN JHJM, KLUIN-NELEMAN HC, KLUIN PM AND SCHUURING E. (1995). Cyclin D1 messenger RNA overexpression as a marker for mantle cell lymphoma. *Oncogene*, **10**, 1833–1840.

DELMER A, AJCHENBAUM-CYMBALISTA F, TANG R, RAMOND S AND FAUSSAT AM. (1995). Over-expression of cyclin D1 in chronic B-cell malignancies with abnormality of chromosome 11q13. *Br. J. Haematol.*, **89**, 798–804.

DULIC V, DRULLINGER LF, LEES E, REED SI AND STEIN GH. (1993). Altered regulation of G1 cyclins in senescent human diploid fibroblasts: accumulation of inactive cyclin E-Cdk2 and cyclin D1-Cdk2 complexes. *Proc. Natl Acad. Sci. USA*, **90**, 11034–11038.

EDWARDS MC AND GIBBS RA. (1994). Multiplex PCR: advantages, development and applications. *PCR Methods Appl.*, **3**, S65–S75.

FREEMAN RS, ESTUS S AND JOHNSON EM. (1994). Analysis of cell cycle-related gene expression in postmitotic neurons: selective induction of cyclin D1 during programmed cell death. *Neuron*, **12**, 343–355.

GILLETT C, FANTL V, SMITH R, FISHER C, BARTEK J, DICKSON C, BARNES D AND PETERS G. (1994). Amplification and overexpression of cyclin D1 in breast cancer detected by immunohistochemical staining. *Cancer Res.*, **54**, 1812–1817.

GINSBERG RJ, KRIS MG AND ARMSTRONG JG. (1993). Cancer of the lung, non-small cell lung cancer. In *Cancer: Principles and Practice of Oncology*, fourth edn., VT de Vita Jr, S Hellman and SA Rosenberg (eds). pp. 673–723. JB Lippincott: Philadelphia.

HAYASHI N, SUGIMOTO Y, TSUCHIYA E, OGAWA M AND NAKAMURA Y. (1994). Somatic mutations of the MTS (Multiple Tumor Suppressor) 1/CDK41 (Cyclin-dependent kinase-4 inhibitor) gene in human primary non-small cell lung carcinomas. *Biochem. Biophys. Res. Commun.*, **202**, 1426–1430.

HEIGHWAY J. (1991). *Hae*III polymorphism within 3'untranslated region of PRAD1. *Nucleic Acids Res.*, **19**, 5451.

HINDS PW, DOWDY SF, EATON EN, ARNOLD A AND WEINBERG RA. (1994). Function of a human cyclin gene as an oncogene. *Proc. Natl Acad. Sci. USA*, **91**, 709–713.

HUNTER T AND PINES J. (1994). Cyclins and cancer II: cyclin D and CDK inhibitors come of age. *Cell*, **79**, 573–582.

JAKOBSEN KS, BREIVOLD E AND HORNES E. (1990). Purification of mRNA directly from crude plant tissues in 15 minutes using magnetic oligo dT microspheres. *Nucleic Acids Res.*, **18**, 3669.

JARES P, FERNANDEZ PL, CAMPO E, NADAL A, BOSCH F, AIZA G, NAYACH I, TRASERRA J AND CARDESA A. (1994). PRAD-1/cyclin D1 gene amplification correlates with messenger RNA overexpression and tumor progression in human laryngeal carcinomas. *Cancer Res.*, **54**, 4813–4817.

JIANG W, KAHN SM, TOMITA N, ZHANG YJ, LU SH AND WEINSTEIN IB. (1992). Amplification and expression of the human cyclin D gene in esophageal cancer. *Cancer Res.*, **52**, 2980–2983.

JIANG W, ZHANG YJ, KAHN SM, HOLLSTEIN MC, SANTELLA RM, LU SH, HARRIS CC, MONTESANO R AND WEINSTEIN IB. (1993). Altered expression of the cyclin D1 and retinoblastoma genes in human esophageal cancer. *Proc. Natl Acad. Sci. USA.*, **90**, 9026–9030.

KANDA Y, NISHIYAMA Y, SHIMADA Y, IMAMURA M, NOMURA H, HIAI H AND FUKUMOTO M. (1994). Analysis of gene amplification and overexpression in human esophageal-carcinoma cell lines. *Int. J. Cancer*, **58**, 291–297.

KAY R AND SCHUMACHER M. (1983). Unbiased assessment of treatment effects on disease recurrence and survival in clinical trials. *Stat. Med.*, **2**, 41–58.

LAMMIE GA, FANTL V, SMITH R, SCHUURING E, BROOKES S, MICHALIDES R, DICKSON C, ARNOLD A AND PETERS G. (1991). D11S287, a putative oncogene on chromosome 11q13, is amplified and expressed in squamous cell and mammary carcinomas and linked to *bcl*-1. *Oncogene*, **6**, 439–444.

LEE JS, RO JY, SAHIN A, HITTELMAN W, BROWN BW, MOUNTAIN CF AND HONG KW. (1989). Expression of epidermal growth factor receptor (EGFR): a favorable prognostic factor for surgically resected non-small cell lung cancer (NSCLC). *Proc. Am. Soc. Clin. Oncol.*, **8**, 226 (A878).

LEW DJ, DULIC V AND REED SI. (1991). Isolation of three novel human cyclins by rescue of $G_1$ cyclin (Cln) function in yeast. *Cell*, **66**, 1197–1206.

LOVEC H, SEWING A, LUCIBELLO FC, MÜLLER R AND MÖRÖY T. (1994). Oncogenic activity of cyclin D1 revealed through cooperation with Ha-*ras*: link between cell cycle control and malignant transformation. *Oncogene*, **9**, 323–326.

LUCIBELLO FC, SEWING A, BRÜSSELBACH S, BÜRGER C AND MÜLLER R. (1993). Deregulation of cyclins D1 and E and suppression of cdk2 and cdk4 in senescent human fibroblasts. *J. Cell Sci.*, **105**, 123–133.

LUKAS J, JADAYEL D, BARTKOVA J, NACHEVA E, DYER MJS, STRAUSS M AND BARTEK J. (1994). BCL-1/cyclin D1 oncoprotein oscillates and subverts the $G_1$ phase control of B-cell neoplasms carrying the t(11;14) translocation. *Oncogene*, **9**, 2159–2167.

LUKAS J, BARTKOVA J, WELCKER M, PETERSON OW, PETERS G, STRAUSS M AND BARTEK J. (1995). Cyclin D2 is a moderately oscillating nucleoprotein required for $G_1$ phase progression in specific cell types. *Oncogene*, **10**, 2125–2134.

MARX J. (1994). How cells cycle toward cancer. *Science*, **263**, 319–321.

MATSUSHIME H, ROUSSEL MF, ASHMUN RA AND SHERR CJ. (1991). Colony-stimulating factor 1 regulates novel cyclins during the $G_1$ phase of the cell cycle. *Cell*, **65**, 701–713.

MICHALIDES R, VAN VEELEN N, HART A, LOFTUS B, WIENTJENS E AND BALM A. (1995). Overexpression of cyclin D1 correlates with recurrence in a group of forty-seven operable squamous cell carcinomas of the head and neck. *Cancer Res.*, **55**, 975–978.

MORI T, MIURA K, AOKI T, NISHIHIRA T, MORI S AND NAKAMURA Y. (1994). Frequent somatic mutation of the MTS1/CDK4I (multiple tumor suppressor/cyclin-dependent kinase 4 inhibitor) gene in esophageal squamous cell carcinoma. *Cancer Res.*, **54**, 3396–3397.

MOTOKURA T AND ARNOLD A. (1993*a*). Cyclin D and oncogenesis. *Curr. Opin. Gen. Develop.*, **3**, 5–10.

MOTOKURA T AND ARNOLD A. (1993*b*). Cyclins and oncogenesis. *Biochim. Biophys. Acta*, **1155**, 63–78.

MOTOKURA T, BLOOM T, KIM HG, JÜPPNER H, RUDERMAN JV, KRONENBERG HM AND ARNOLD A. (1991). A novel cyclin encoded by a *bcl*1-linked candidate oncogene. *Nature*, **350**, 512–515.

MÜLLER D, MILLON R, LIDEREAU R, ENGELMANN A, BRONNER G, FLESCH H, EBER M, METHLIN G AND ABECASSIS J. (1994). Frequent amplification of 11q13 DNA markers is associated with lymph node involvement in human head and neck squamous cell carcinomas. *Eur. J. Cancer B. Oral Oncol.*, **30**, 113–120.

MUSGROVE EA, LEE CSL, BUCKLEY MF AND SUTHERLAND RL. (1994). Cyclin D1 induction in breast cancer cells shorten G1 and is sufficient for cells arrested in G1 to complete the cell cycle. *Proc. Natl Acad. Sci. USA*, **91**, 8022–8026.

NAKAMURA S, SETO M, BANNO S, SUZUKI S, KOSHIKAWA T, KITOH K, KAGAMI Y, OGURA M, YATABE Y. AND KOJIMA M. (1994). Immunohistochemical analysis of cyclin D1 protein in hematopoietic neoplasms with special reference to mantle cell lymphoma. *Jpn. J. Cancer Res.*, **85**, 1270–1279.

NELSON W. (1969). Hazard plotting for incomplete failure data. *J. Qual. Technol.*, **1**, 27–52.

NISHIDA N, FUKUDA Y, KOMEDA T, KITA R, SANDO T, FURUKAWA M, AMENOMORI M, SHIBAGAKI I, NAKAO K, IKENAGA M AND ISHIZAKI K. (1994). Amplification and overexpression of the cyclin D1 gene in aggressive human hepatocellular carcinoma. *Cancer Res.*, **54**, 3107–3110.

OTTERSON GA, KRATZKE RA, COXON A, KIM YW AND KAYE FJ. (1994). Absence of p16INK4 protein is restricted to the subset of lung cancer lines that retains wildtype Rb. *Oncogene*, **9**, 3375–3378.

PETERS G. (1994). The D-type cyclins and their role in tumorigenesis. *J. Cell Sci. Suppl.*, **18**, 89–96.

PEZZELLA F, TURLEY H, KUZU I, TUNGEKAR MF, DUNNILL MS, PIERCE CB, HARRIS A, GATTER KC AND MASON DY. (1993). BCL-2 protein in non-small-cell lung carcinoma. *N. Engl. J. Med.*, **329**, 690–694.

PINES J. (1993). Cyclins and their associated cyclin-dependent kinases in the human cell cycle. *Biochem. Soc. Trans.*, **21**, 921–925.

QUELLE DE, ASHMUN RA, SHURTLEFF SA, KATO JY, BAR-SAGI D, ROUSSEL MF AND SHERR CJ. (1993). Overexpression of mouse D-type cyclins accelerates $G_1$ phase in rodent fibroblasts. *Genes Dev.*, **7**, 1559–1571.

SCHUURING E, VERHOEVEN E, VAN TINTEREN H, PETERSE JL, NUNNINK B, THUNNISSEN FBJM, DEVILEE P, CORNELISSE CJ, VAN DE VIJVER M, MOOI WJ AND MICHALIDES RJAM. (1992). Amplification of genes within the chromosome 11q13 region is indicative of poor prognosis in patients with operable breast cancer. *Cancer Res.*, **52**, 5229–5234.

SERRANO M, HANNON GJ AND BEACH D. (1993). A new regulatory motif in cell-cycle control causing specific inhibition of cyclin D/CDK4. *Nature*, **366**, 704–707.

SETO M, YAMAMOTO K, IIDA S, AKAO Y, UTSUMI KR, KUBONISHI I, MIYOSHI I, OHTSUKI T, YAWATA, Y, NAMBA M, MOTOKURA T, ARNOLD A, TAKAHASHI T AND UEDA R. (1992). Gene rearrangement and overexpression of PRAD1 in lymphoid malignancy with t(11;14)(q13;q32) translocation. *Oncogene*, **7**, 1401–1406.

SEWING A, BÜRGER C, BRÜSSELBACH S, SCHALK C, LUCIBELLO FC AND MÜLLER R. (1993). Human cyclin D1 encodes a labile nuclear protein whose synthesis is directly induced by growth factors and suppressed by cyclic AMP. *J. Cell Sci.*, **104**, 545–554.

SHAPIRO GI, EDWARDS CD, KOBZIK L, GODLESKI J, RICHARDS W, SUGARBAKER DJ AND ROLLINS BJ. (1995). Reciprocal Rb inactivation and p16INK4 expression in primary lung cancers and cell lines. *Cancer Res.*, **55**, 505–509.

SHERR CJ. (1993). Mammalian G1 cyclins. *Cell*, **73**, 1059–1065.

SHERR CJ. (1994). G1 phase progression cycling on cue. *Cell*, **79**, 551–555.

SHERR CJ. (1995). D-type cyclins. *Trends Biochem. Sci.*, **20**, 187–190.

*UICC-AMERICAN JOINT COMMITTEE ON CANCER.* (1987). *Manual for Staging of Cancer*. 3rd edn. JB Lippincott: Philadelphia.

WHO (1981). Histological Typing of Lung Tumours, 2nd edn. World Health Organization: Geneva.

WILLIAMS ME, GAFFEY MJ, WEISS LM, WILCZYNSKI SP, SCHUURING E AND LEVINE PA. (1993). Chromosome 11q13 amplification in head and neck squamous cell carcinoma. *Arch. Otolaryngol. Head Neck Surg.*, **119**, 1238–1243.

WIMAN KG. (1993). The retinoblastoma gene: role in cell cycle control and cell differentiation. *FASEB J.*, **7**, 841–845.

WITHERS DA, HARVEY RC, FAUST JB, MELNYK O, CAREY K AND MEEKER TC. (1991). Characterization of a candidate bcl-1 gene. *Mol. Cell Biol.*, **11**, 4846–4853.

XIONG Y, CONNOLLY T, FUTCHER B AND BEACH D. (1991). Human D-type cyclin. *Cell*, **65**, 691–699.

ZHANG SY, KLEIN-SZANTO AJP, SAUTER ER, SHAFARENKO M, MITSUNAGA S, NOBORI T, CARSON DA, RIDGE JA AND GOODROW TL. (1994). Higher frequency of alterations in the p16/CDKN2 gene in squamous cell carcinoma cell lines than in primary tumors of the head and neck. *Cancer Res.*, **54**, 5050–5053.

ZHANG YJ, JIANG W, CHIEN CJ, LEE CS, KAHN SM, SANTELLA RM AND WEINSTEIN IB. (1993). Amplification and overexpression of cyclin D1 in human hepatocellular carcinoma. *Biochem. Biophys. Res. Commun.*, **196**, 1010–1016.

ZHOU X, TARMIN L, YIN J, JIANG HY, SUZUKI H, RHYU MG, ABRAHAM JM AND MELTZER SJ. (1994). The MTS1 gene is frequently mutated in primary human esophageal tumors. *Oncogene*, **9**, 3737–3741.